# Supplementary material for: Granular analysis of pathways to care and durations of untreated psychosis: A marginal delay model
Source: PLoS One. 2022 Dec 6;17(12):e0270234. doi: 10.1371/journal.pone.0270234 (PMC9725156; doi:10.1371/journal.pone.0270234)
Supplement: S1 Table — The unadjusted model tests for correlation of individual predictors with the dependent variable marginal-delay. The adjusted or multivariate model test for correlation of each predictor when other predictors occur. (DOCX) [file pone.0270234.s001.docx]

**Table 5.** Mixed model repeated measure analysis of effect of patient characteristics on **Marginal-delay** as outcome measure. The unadjusted model tests for correlation of individual predictors with the dependent variable Marginal-delay. The adjusted or multivariate model test for correlation of each predictor when other predictors occur.

|  | **Unadjusted model** | P value | **Multivariate model** | P value |
| --- | --- | --- | --- | --- |
| **Node type** |  | **<.0001** |  | **<.0001** |
| Inpatient | 19.5 (3.3, 35.7) |  | 18.2 (-4.1, 40.6) |  |
| Outpatient | 141.6 (119.5, 163.8) |  | 135.9 (109.6, 162.1) |  |
| ED | 2.4 (-12.9, 17.7) |  | 2.0 (-19.7, 23.6) |  |
| **Age (enrollment)**, year | -0.02 (-2.7, 2.6) | 0.9873 | -0.8 (-3.2, 1.7) | 0.5402 |
| **Age (onset)**, year | -1.7 (-4.4, 0.9) | 0.1905 |  |  |
| **Sex** |  | 0.8772 |  | 0.7286 |
| Female | 39.5 (19.5, 59.4) |  | 50.1 (26.4, 73.9) |  |
| Male | 37.6 (25.6, 49.7) |  | 53.9 (36.7, 71.1) |  |
| **Race** |  | 0.1204 |  | 0.2444 |
| White | 45.0 (25.5, 64.5) |  | 49.5 (27.9, 71.1) |  |
| Black | 47.2 (31.6, 62.7) |  | 66.4 (49.6, 83.3) |  |
| Interracial | 19.8 (-11.9, 51.5) |  | 32.3 (3, 61.5) |  |
| Hispanic | 15.8 (-7.5, 39.2) |  | 48.7 (24.5, 72.9) |  |
| Other | 69.1 (-0.9, 139) |  | 63.2 (-6.1, 132.6) |  |
| **Income** |  | 0.1548 |  | 0.4950 |
| Less than $39,999 | 30.4 (14.3, 46.5) |  | 46.0 (23.2, 68.8) |  |
| $40,000 to $59,999 | 29.3 (4.3, 54.3) |  | 40.6 (12.1, 69) |  |
| $60,000 to $99,999 | 31.0 (6.4, 55.5) |  | 45.3 (21.1, 69.5) |  |
| $100,000 and above | 62.4 (38.3, 86.6) |  | 65.2 (36.9, 93.5) |  |
| Don’t know or refused | 56.1 (22.4, 89.9) |  | 63 (27.9, 98.1) |  |
| **GAF-e** | 0.4 (-0.5, 1.3) | 0.3631 | 0.3 (-0.5, 1.1) | 0.4612 |
| **GAF-12** | -1.1 (-1.6, -0.5) | **0.0005** | -0.7 (-1.3, -0.1) | **0.0139** |
| **GAF- Δ** | 0.9 (0.4, 1.4) | **0.0006** |  |  |

GAF-e : Global Assessment of Functioning, the month before enrollment

GAF-12: GAF 12 months prior to enrollment in clinic.

GAF-Δ : arithmetic difference between GAF-e and GAF-12
