# Supplementary material for: Granular analysis of pathways to care and durations of untreated psychosis: A marginal delay model
Source: PLoS One. 2022 Dec 6;17(12):e0270234. doi: 10.1371/journal.pone.0270234 (PMC9725156; doi:10.1371/journal.pone.0270234)
Supplement: Attachment — Submitted filename: Reviewer Comments.docx [file pone.0270234.s003.docx]

Response to Reviewers,

We appreciate the close reading and thoughtful comments by editorial staff and both reviewers. Below you will find an item-by-item response to each recommendation.

When submitting your revision, we need you to address these additional requirements.

1. Please ensure that your manuscript meets PLOS ONE's style requirements, including those for file naming. The PLOS ONE style templates can be found at 
<https://journals.plos.org/plosone/s/file?id=wjVg/PLOSOne_formatting_sample_main_body.pdf> and 
<https://journals.plos.org/plosone/s/file?id=ba62/PLOSOne_formatting_sample_title_authors_affiliations.pdf>

Made changes outlined in style requirements

3. Have the authors made all data underlying the findings in their manuscript fully available?

The [PLOS Data policy](https://nam12.safelinks.protection.outlook.com/?url=http%3A%2F%2Fwww.plosone.org%2Fstatic%2Fpolicies.action%23sharing&data=04%7C01%7Cwalter.mathis%40yale.edu%7Cfaceb8c7237949198c0e08d9ec51b52f%7Cdd8cbebb21394df8b4114e3e87abeb5c%7C0%7C0%7C637800659012568272%7CUnknown%7CTWFpbGZsb3d8eyJWIjoiMC4wLjAwMDAiLCJQIjoiV2luMzIiLCJBTiI6Ik1haWwiLCJXVCI6Mn0%3D%7C3000&sdata=GIsrJnCugxpQIbrWdzzHd494VbNr0FNdzdv4fQiiAYg%3D&reserved=0) requires authors to make all data underlying the findings described in their manuscript fully available without restriction, with rare exception (please refer to the Data Availability Statement in the manuscript PDF file). The data should be provided as part of the manuscript or its supporting information, or deposited to a public repository. For example, in addition to summary statistics, the data points behind means, medians and variance measures should be available. If there are restrictions on publicly sharing data—e.g. participant privacy or use of data from a third party—those must be specified.

Reviewer #1: No

Reviewer #2: No

Will update our data availability statement to declare “data contain potentially identifying or sensitive patient information”.

5. Review Comments to the Author

Please use the space provided to explain your answers to the questions above. You may also include additional comments for the author, including concerns about dual publication, research ethics, or publication ethics. (Please upload your review as an attachment if it exceeds 20,000 characters)

Reviewer #1: Strengths of the paper: Well justified background. Data from a well-established and methodologically sound programme for first episode psychosis. Conceptual model is very well developed. The network node analysis is particularly interesting and novel – it certainly adds value to the field as it indicates likely nodes that both affect PTC and delay. The functioning results are also very interesting.
The issues below require addressing in a revised version – for the most part these should be straightforward to do. My most substantive comment is the last – I think there needs to be some substantive reworking of the discussion to give credence to some possible limitations of the work. Other areas of the discussion could be shortened (particularly the initial summary) to keep the good flow and precision which this work otherwise exhibits.

1. In general, take care throughout in using the term “significant”. It should generally be reserved to denote a statistically significant result (with test statistics reported), with another word used to describe other important findings. i.e. P12 “However, a significant number of Clinical nodes interacted with FEP patients but did not appear to recognize psychosis.” Here this is interesting, but perhaps “substantial” is a more appropriate qualifier – make sure to back up such claims with the actual data.
Noted and clarified.

2. Please report IQR alongside any medians reported in the paper (including abstract).

Noted and done

3. Please report the type of statistical tests used when reporting p-values.

Noted and done

4. In abstract, first sentence of results could be deleted, or values that are reported in second sentence could be integrated into first sentence, to shorten. Report how much longer (in days) the DUP was for those with sharper functional decline.

Noted and done

5. P5, preference to use First Episode Psychosis [FEP] services as the nomenclature to distinguish this type of specialism from first episode programmes for other disorders, and more consistently used in the (at least European) literature.

Noted and done

6. P6, methods: When was the GAF measured on participants? This is stated on p9, but I recommend moving it to where this is first introduced. More detail of how GAF 12 months prior to STEP enrolment should be given.

Clarified

7. P8, methods: I recommend removing “age at STEP enrolment” from the analyses and paper. This cannot influence delay, and any associations here will be uninformative because they will be perfectly correlated with age at symptom onset and DUP.

Noted and done

8. P9, please report the corresponding statistical test and p-values for the statement “These participants did not differ significantly from the remaining subjects in age, race, or gender.”

Noted and done

9. P13, Table 2 – it’s unclear from the table whether the correlations are for DUP-total to total nodes, DUP-demand to Demand nodes, DUP-supply to Supply nodes. I assume this is the case, but this should be clarified in the table, and text on P12.

Clarified this relationship graphically in Table and updated language in text (p13)

10. P13, Table 3: Please retitle the table to avoid the use of the word “correlations” – technically I think only the continuous-continuous associations are correlations; associations is preferred.

Noted and done

11. P16: For consistency it is probably easier to report the median ED encounters per participant than the mean number (with IQR).

Noted and done

12. Discussion: repetition of results (i.e. values) should be avoided in the discussion.

Noted and done

13. Discussion: a separate “strengths and limitations” section should be provided with a subheading. In particular, the paper should discuss:

a. The extent to which data collected in their study (i.e. the reconstruction of PTC) was reliant on clinical records versus patient and caregiver reports. The former are collected prospectively, and thus minimise recall, whereas the latter may be affected by recall, which may act differentially – particularly by level of functioning which could have the potential to bias the results. If the authors have data on the proportions of PTC records rated from these sources, this would be helpful additional information; if this was not recorded, a more detailed discussion on this general point will suffice.

This is a good point and included in the limitations section. Our PTC reconstruction process did not allow us to subset sources of data in any meaningful way (to determine proportion of clinical records vs patient and caregiver report).

b. The validity of defining DUP-supply as the date from the initiation of antipsychotics. It is possible for clinical contacts before this point to “fail” to prescribe Aps, which could (retrospectively) be seen as a supply-side delay in treatment. Alternatively, is a clinical contact (without Aps) a possible treatment in itself – i.e. if they received psychological interventions at this point or some other form of active monitoring?

Indeed, while we feel strongly that AP is a pragmatic and reliable way to index the transition from demand to supply, it is certainly imperfect and there are feasible cases where it might fail to detect the transition. We attempted address this in the explanation of the methods and the limitations section of the discussion.

c. Detection bias – the sample is, by definition, restricted to those who are referred to and enrolled in STEP. There may be other FEP cases in the community (i.e. the true incidence) that have not yet been referred to STEP. Although the extent of this is unknown, a comment on it is worthwhile – in the true population with FEP in the STEP catchment, could it be that the true DUP was even longer than observed here.

Comment added.

d. Given the high proportion of “false positive” referrals to STEP (i.e. 199/1356 enrolled) could STEP be a significant supply-side node contributing to marginal delays for people with other (non-psychotic) psychopathologies that require onward referral to other specialised mental health programmes (where available)?

This is a fair point. Our focus, both in the paper and in STEP, is on non-affective psychosis. But, we don’t think STEP was a significant supply-side delay for the 1157/1356 who were referred but not appropriate for STEP because 1) STEP evaluation happened very quickly (within 1-2 days) and 2) inappropriate referrals were always provided with information for alternative, more appropriate clinics. Comment added, combined with detection bias point above.

e. Could the absence of variation in delays by demographic characteristics be a function of statistical power and the small sample size? Table 3 only reports P-values, but could more helpfully report effect sizes / differences between the comparator groups.

Agree that the small n limits strong statistical inferences. Per request have included eta2 and rhos for all associations/correlations in Table 3 to better understand effects size.

**Reviewer #2:** The authors have developed an approach to analyze delays in the pathways to care for patients with psychosis. They developed and tested this method on data from 156 participants enrolled in an early treatment program. This proof of concept on a specific sample provides insight into the associations between certain events and delayed care, and is an interesting approach to quantify and analyze pathways to care. The manuscript could benefit from clarifying the assumptions and approach so that the importance become more clear.

Abstract:
From the information in the abstract, it is not immediately clear what “Community nodes” are. The abstract should be interpretable without having to read the manuscript.

Noted and done

After reading the manuscript, I think it could be clarified partially by defining the antipsychotic prescription as the switch from demand to supply.

Removed this language from the abstract for clarity and brevity

“referred to the clinic” -> does this mean STEP?

Noted and clarified

Choose “v” or “vs” when comparing two items, not both.

Noted and done

It seems like the words community, clinical, outpatient programs don’t need to be capitalized, as they’re used as-is in this context, and it makes it harder to read. Later on in the manuscript, the same applies to marginal delay. I’m still on the fence regarding Demand and Supply, but see comments in introduction and methods on using these terms in general.

Noted and done

Regardless, without defining Demand and Supply, these terms are currently meaningless in the abstract.

Agree and reworded abstract to be independent of these proprietary terms.

The manuscript only provides “actionable insights on how to focus efforts on strategies to reduce untreated psychosis”, but not “how to best measure or analyze strategies”; Only one approach is analyzed in the manuscript and so there is no head-to-head analytical (nor descriptive) comparison of this approach with other methods. Removed “best”

Furthermore, what are the actionable insights? Please state that instead of just mentioning that there are actionable insights.

Clarified this language. While actionable insights are included in the discussion, it was confusing in this sense.

Introduction:
The paragraph “The concept of pathways…” hints at prior studies that dissect pathways to care (beyond just measuring the DUP), but some specifics would be good to have here. It is not immediately clear that this study will be any different from the prior literature; why would it not have the same shortcomings? In this paragraph, the most recent citation is from 2014 – has there been no more efforts in this field after this year?

Updated citations to clarify this point

The PTC of patients is complex, multifaceted and heterogeneous, and I commend the authors for attempting to create a general measure that captures all aspects. The assumptions of the approach, however, could use some clarification. The following paragraph “From an interventionist perspective…” is not defining the treatment. Later it becomes clear that this treatment event only consists of being prescribed antipsychotic prescription drugs.

Sorry if this was confusing – but we feel that “treatment” in this case is defined as “entry into specialty team-based First Episode Services” – as described in the end of the ‘Supply’ side.

Why exactly is anti-psychotic prescription the defining moment where it switches from demand to supply? As DUP could be defined in multiple ways, the authors may wish to expand this section to provide the underpinnings of what is considered the end of the DUP (i.e. why would only STEP and not any other treatments be considered the start of the treatment). What if the patient receives outpatient care from a psychiatrist (with or without medication) before being enrolled in STEP, wouldn’t this be considered the end of the untreated period, especially if STEP wasn’t an option? Some of these details may be better suited for the methods section, but overall, more justification is needed for the general approach, and why it is better than everything else that has been published before. If the authors’ main purpose is provide a proof of concept, and not derive any conclusions from the content because of these assumptions, this should be more clearly mentioned.

Appreciate thoughtful notes. We have added significant language to justify (and then admit limitation of) antipsychotic prescription as liminal event. And, to the second point, STEP enrollment was considered the end event because this was specifically an analysis of PTCs to enrollment in first episode services in the context of an early detection campaign with the goal of treating all first episode services in out catchment area. It was also intended to outline an analytic approach for other FEP clinics to use to evaluate their local healthcare network and recruitment efforts to get first episode cases quickly to their specialized care. We will update some relevant language in the paper to make this clearer.

Methods:
What is the size and type of catchment area of STEP, is it rural, urban, suburban etc?

Noted and clarified

Were there any differences in recall rates between the place of care? E.g. an emergency room visit is more memorable than perhaps mentioning symptoms at a yearly physical exam.

We do not have data available to comment on this – though it highlights the recall bias of our data which we further explored in the limitations section.

Enrolment should be enrollment (also in legend of Table 1, and discussion).
From the examples in the legend of figure 1, it becomes somewhat clear what kind of agents define a community node and clinical node, but these terms should be explained in the text of the methods with a comprehensive definition (or a list in the supplement of all the possible types of events that were mentioned by the patients). Furthermore, the authors later on make a distinction between community and clinical nodes that, later on, appears to be a critical concept in the analysis. The importance should be described in the introduction. Clarified the introduction of these terms.

Related to a comment on the introduction - what happens when the patient does not get, want, need, or take anti-psychotics? Especially considering the younger age of the sample, anti-psychotics may not be a desirable intervention for all patients.

This is valid point and perhaps weakness underlying our use of antipsychotic prescription as liminal event supply and demand and we added some discussion of this to our discussion section. While imperfect, we still feel that the prescription of an antipsychotic for psychosis is one of the strongest indications of the recognition of psychotic illness that can be assessed in a retrospective approach.

If they get only a one-month fill of anti-psychotics during the DUP period, and don’t get a refill, according to the current definition, shouldn’t they switch back from supply to demand?

Regarding this particular case, using our formulation we do not feel that we would then switch back from supply to demand. In our formulation, the switch from demand to supply happens when psychotic illness has been recognized by a clinical provider and from that point forward it is the onus of the clinical treatment network, broadly conceptualized, to get the patient quickly and directly to specialized treatment. The patient stopping meds after one month does not change this.

Did the patients provide information on when they had an unmet need of care, or whether symptoms were manageable without care during the DUP period?

This is a fair point and added to the limitations section. We did not collect such information from the participants.

Would it be possible to inquire with the participants to find out at what stages they considered to be in the demand/supply period? I am not necessarily dismissing the assumptions that are admittedly hard to define and generalize among all patients, but it appears that the authors are making generalized decisions about the patients’ needs without their direct input. With the current level of detail it is hard to determine whether that is a reasonable approach to the problem.

Unfortunately, collection of such data is no longer possible as the majority of participants have timed out of the STEP clinic and we are no longer in contact / collecting data from them.

I am now aware that other papers describing STEP have used the demand/supply terms, but it appears that these words were defined differently: ‘Demand’: identification of illness and initiation of help-seeking; and ‘Supply’: correct identification of diagnosis and referral to first-episode services (from Srihari et al, BMC Psychiatry 2014). Perhaps a better choice of words in this context would be pre-medication/post-medication. This would also make it clear to the readers (who may not read the methods carefully) that that event is defined as the switch. Or a different approach would be to follow the original definition.

Appreciate the thoughtful eye toward consistent terminology for STEP as this is something we take quite seriously as well. The use of AP as the transition from demand to supply has been used internally at STEP for several years, see for example reference 36 (Srihari, Ferrara, et al 2022). As discussed above (and expanded on revision to this paper) we in fact do feel that AP is a reasonable, if not perfect, signifier of the transition from epoch of PTC that is delay in illness identification and delayed attempts to access healthcare (demand) and delays by professionals to initiate treatment including delays in diagnosis and initiating or referring to appropriate treatment (supply).

Figure 1 shows a very short DUP, considering that the median DUP in the study sample was 151 days. What was the range? IQR is included in Table 2.

It would be more representative to show a longer DUP in the figure, or at the very least acknowledge in the legend that the duration is short for illustrative purposes.

This note was added to the figure description.

If allowed for privacy reasons, I think it would be interesting if the timelines for all or a representative subset of patients could be shown in a figure (probably in a supplemental section), since there are only 156 patients. This would give a better idea of the distribution of DUP time, and the sequences and kind of nodes that occurred in the whole sample. For researchers who wish to apply the approach developed by the authors, this may give a better idea of whether it would be an informative and feasible approach for their own group of patients, especially considering the general heterogeneity of patient samples and enrollment in a specialized treatment program in this study. The timeline depicted in Fig 1 was drawn by hand. While our n is only 156, there are over 1100 nodes across all PTCs. While we agree to the theoretical interest of such supplementary data, we believe drawing (or constructing a software program to automatically draw) all 156 PTCs is an overly burdensome task beyond the scope of this revision.

A delay is described as the days after the event until the next. However, the counterfactual is not known. A community node may slow down or speed up the next event if the event had not occurred, but this is unobserved. How do the authors reconcile not knowing the counterfactual?

I am not 100% sure that I understand the reviewer’s point here, but I will attempt to address my interpretation of the question. At the most fundamental level, the only data available for a PTC are nodes on the pathway and when they occurred. While we cannot even know all permutations and eventualities that diverge exponentially from every node step. With such perfect (but impossible) knowledge, we could likely draw more definitive, causal associations between delay from node actually travelled (compared to all others not). But, barring that, and with noted limitations to the strength of delay causality, we still think the approach is the best with the given data for subdividing delay by node constituents. In purest terms: in this retrospective approach we have defined the starting point as the onset of illness and the destination enrollment into STEP. Everything between those two points account for the overall delay and marginal delay is the soundest approach to identify where the subdivisions of delay started – and hence where, in the case of longer delays, interventions should be focused to reduce it.

A few more details on the mixed model methods would be helpful.

Noted and done

The authors would be encouraged to share their R and SAS code used for the analyses to enable other researchers to apply their approach.

Noted and done

Results
The word note in the “Network” section should be node.

Noted and done

The results in the sentence “Unsurprisingly, the majority…” are not surprising because they’re in part mechanical. It might be good to add this, for those who did not read the methods carefully. Similarly, the sentence “While community node encounters skewed toward the demand side” later on in the Node category section, may be clarified.

Noted and done

In the sentence “However, a significant number of clinical nodes…” it would be good to quantify this in some way.

Noted and done

The sentence “Interactions with police…” makes it initially seem like the authors combined police and self-referral while these are very different from each other. After inspecting the table, it appears that it’s just the percentages that are the same. This could be clarified with writing “(40% of participants in each category)” or something similar.

Noted and done

“… the relative frequency of encounters per node type…” doesn’t appear stable -as mentioned to a certain extent- as the self category is higher, and all the other categories are lower. Perhaps the sentence should read: The distribution of the community node types changed between the demand and supply time period, with ‘self’ occurring relatively more frequently, and the other node types occurring less frequently on the supply side.

Noted and done

In general, delays seem to be causally attributed to nodes in this manuscript, but causality cannot be inferred from these methods . Throughout the text the wording (e.g. “contribute” to delay, or making the jump to delay-reducing interventions) should be changed to reflect that

While we intended the “contribution” in a mathematical sense, we agree that it might be confusing and have updated language.

Discussion:
The results don’t necessarily “illustrate robustness of the approach”. I didn’t see any sensitivity analyses, which is acceptable for the purposes of this manuscript, but this sentence should probably be left out.

Noted and done

The results also don’t “validate the analytical approach”. The results are very interesting and informative from an explorative viewpoint, but the authors should be careful not to overstate the conclusions and implications of the study.

Noted and done

First and foremost, more research is needed before it is clear that this method would be equally informative with other patients or in other contexts (e.g. no enrollment in the STEP program). An explanation of why the results may not generalize would be helpful.

Thank you for this point and language addressing this was added to the limitations section.

The structure of the sentence “This validates the utility of multi-component ED campaigns…” could be improved.

A sensitivity analysis that includes the partially known PTCs of currently excluded participants may be informative, as in other contexts it is likely not clear whether PTCs are complete or not.

This is an interesting point. Unfortunately, when a PTC was deemed incomplete or internally inconsistent (eg two events dated in illogical sequence) they were not digitally transcribed and their data is not available for re-analysis. This point also highlights how our analytic technique depends on a complete PTC as delay inferences are made from this assumption. This is certainly a potential limitation in some settings and will be added to the limitations section of the discussion.

Conclusion
See comments regarding the conclusion in the abstract section.
